# Prediction of Microvascular Invasion in Hepatocellular Carcinoma With a Multi-Disciplinary Team-Like Radiomics Fusion Model on Dynamic Contrast-Enhanced Computed Tomography

**DOI:** 10.3389/fonc.2021.660629

**Published:** 2021-03-16

**Authors:** Wanli Zhang, Ruimeng Yang, Fangrong Liang, Guoshun Liu, Amei Chen, Hongzhen Wu, Shengsheng Lai, Wenshuang Ding, Xinhua Wei, Xin Zhen, Xinqing Jiang

**Affiliations:** ^1^ Department of Radiology, Guangzhou First People’s Hospital, Guangzhou Medical University, Guangzhou, China; ^2^ Department of Radiology, Guangzhou First People’s Hospital, School of Medicine, South China University of Technology, Guangzhou, China; ^3^ School of Biomedical Engineering, Southern Medical University, Guangzhou, China; ^4^ School of Medical Equipment, Guangdong Food and Drug Vocational College, Guangzhou, China; ^5^ Department of Pathology, Guangzhou First People’s Hospital, School of Medicine, South China University of Technology, Guangzhou, China

**Keywords:** hepatocellular carcinoma, microvascular invasion, dynamic contrast-enhanced computed tomography, radiomics, model fusion

## Abstract

**Objective:**

To investigate microvascular invasion (MVI) of HCC through a noninvasive multi-disciplinary team (MDT)-like radiomics fusion model on dynamic contrast enhanced (DCE) computed tomography (CT).

**Methods:**

This retrospective study included 111 patients with pathologically proven hepatocellular carcinoma, which comprised 57 MVI-positive and 54 MVI-negative patients. Target volume of interest (VOI) was delineated on four DCE CT phases. The volume of tumor core (V*_tc_*) and seven peripheral tumor regions (V*_pt_*, with varying distances of 2, 4, 6, 8, 10, 12, and 14 mm to tumor margin) were obtained. Radiomics features extracted from different combinations of phase(s) and VOI(s) were cross-validated by 150 classification models. The best phase and VOI (or combinations) were determined. The top predictive models were ranked and screened by cross-validation on the training/validation set. The model fusion, a procedure analogous to multidisciplinary consultation, was performed on the top-3 models to generate a final model, which was validated on an independent testing set.

**Results:**

Image features extracted from V*_tc_*+V*_pt(12mm)_* in the portal venous phase (PVP) showed dominant predictive performances. The top ranked features from V*_tc_*+V*_pt(12mm)_* in PVP included one gray level size zone matrix (GLSZM)-based feature and four first-order based features. Model fusion outperformed a single model in MVI prediction. The weighted fusion method achieved the best predictive performance with an AUC of 0.81, accuracy of 78.3%, sensitivity of 81.8%, and specificity of 75% on the independent testing set.

**Conclusion:**

Image features extracted from the PVP with V*_tc_*+V*_pt(12mm)_* are the most reliable features indicative of MVI. The MDT-like radiomics fusion model is a promising tool to generate accurate and reproducible results in MVI status prediction in HCC.

## Introduction

Hepatocellular carcinoma (HCC), is the fifth most common cancer and third leading cause of cancer-related death worldwide (1). Despite advancements in medical technology leading to great achievements in the treatment of HCC, the prognosis of HCC remains poor, with a 5-year recurrence rate of 70% with hepatectomy and 35% following liver transplantation (2–4). Microvascular invasion (MVI) is defined as the presence of tumor cells in portal veins, large capsule vessels, or in the vascular space lined by endothelial cells (5, 6). Evidence has shown that MVI is an independent predictor of recurrence and poor outcome following surgical hepatic resection (4, 7–9).

Currently, pathological examination is the gold standard for identifying MVI of HCC following operation or biopsy collection. This approach, however, is unreliable in case of sample contamination or for ineffectively done preoperative needle biopsies due to intratumoral heterogeneity (10). Furthermore, needle biopsy may increase the risks of unintended tumor bleeding or implantation metastasis (11). Therefore, there is a pressing demand for an accurate and non-invasive method for early prediction of MVI.

To date, the identified clinical indicators of MVI include: Alpha-fetoprotein (AFP), prothrombin induced by vitamin K absence or antagonist II (PIVKA-II), and other serological markers (12). However, the prediction performances of these markers were unsatisfactory due to poor sensitivity and specificity. Morphological and imaging characteristics such as large tumor size, unsmooth tumor margins, multinodular tumor morphology, rim enhancement on the arterial phase, and peritumoral hypointensity on the hepatobiliary phase of Gadolinium-exthoxybenzyl-diethylenetriamine-pentaacetic (Gd-EOB-DTPA)–enhanced MRI, have also been associated with the presence of MVI (13–18). These characteristics, on the other hand, are easily prone to inter-observer variations (19), as evidenced by the inconsistent interpretations of the conventional CT/MRI images for MVI prediction in previous studies (20, 21).

Radiomics is a new method for disease diagnosis and prognosis prediction. Radiomics have exhibited great potential in predictive/discriminative models by integrating disease-related imaging features with clinical, pathological, and genetic data (22–24). Progress has been made in applying CT or MRI based radiomics for investigating MVI in HCC (25–28). However, most studies only included a single phase of dynamic contrast-enhanced (DCE)-CT or MRI. Exclusion of the other phases is likely to omit useful information which may impair MVI prediction. Also, research has focused mainly on the intratumoral region. Therefore, key image information beyond the tumor core might be lost since MVI often occurs in regions neighboring the tumor/non-tumor interface (6, 29). Image texture from the peripheral liver parenchyma, such as a settled 5mm or 10mm distance from the tumor edge, have shown encouraging predictive ability in MVI (26, 28, 30). To our knowledge, no study has comprehensively reported on MVI predictive performances using different combinations of DCE-CT/MRI phase(s) with varying distances from the tumor margin.

Predictive performance is also closely related to the prediction model (or classifier) used. Different classifiers are built on different mathematical models and thus generate inconsistent performances with the same classification task (31). An ensemble of classifiers, a process analogous to disease diagnosis by a multi-disciplinary team (MDT), produces more reliable and accurate predictions compared with a single classifier (32–34). This study hypothesizes that this principle also applies with the fusion of various predictive models to yield enhanced and reproducible performance in MVI prediction.

The main study objective was to investigate the performance of radiomics analysis for MVI prediction in HCC. This study also aimed to identify the predominant phase(s) and the most relevant tumor periphery range for MVI prediction using noninvasive DCE-CT. Miscellaneous predictive models are established by considering different combinations of phases, tumor peripheral margins, feature selection methods and classifiers. The predictive performances of each model, as well as the final fusion model obtained through a multi-disciplinary team (MDT)-like fusion method, were to be explored.

## Materials and Methods

### Patient Cohort

This study was approved by the Institutional Review Board of Guangzhou First People’s Hospital and the requirement for informed consent was waived based on the nature of a retrospective study. A total of 212 patients who underwent preoperative DCE-CT for newly diagnosed HCC from January 2016 to April 2020 at Guangzhou First People’s Hospital were considered for inclusion in the study. The inclusion criteria were: 1) Pathologically confirmed HCC; 2) preoperative quadriphasic DCE-CT performed, and 3) complete preoperative lab tests. The exclusion criteria were: 1) Patients who had received anticancer therapy including chemoembolization, radiofrequency ablation, or transcatheter arterial chemoembolization (*n*=98); and 2) time interval between DCE-CT scan and surgery of more than two weeks (*n*=3). Finally, a total of 111 HCC patients (MVI positive: *n*=57 and MVI negative: *n*=54) were enrolled in this study.

The clinical considerations included: Presence or absence of cirrhosis, hepatitis B or C immunology. While the preoperative tests carried out included: alpha-AFP level, white blood cell (WBC) count, red blood cell (RBC) count, neutrophil count, hemoglobin (Hb) level, serum albumin (ALB), platelet count (PLT), prothrombin time (PT), international normalized ratio (INR), aspartate aminotransferase (AST), serum alanine aminotransferase (ALT), conjugated bilirubin (CB), serum total bilirubin (TB), serum creatinine (Scr), serum alkaline phosphatase (ALP), and determination of Child-Pugh class.

### Imaging and Histopathology

Preoperative DCE-CT were performed on multiple scanners with four phases following intravenous injection of the contrast agent, including phase 1- early arterial phase (EAP), 18-25 s; phase 2- late arterial phase (LAP), 35-40 s; phase 3- portal venous phase (PVP), 50-60 s; and phase 4- equilibrium phase (EP) 120-250 s. The detailed imaging parameters are shown in the **Supplementary Materials**.

All surgical specimens were examined by one pathologist (W.S. Ding, with 14 years of experience in pathological diagnosis of hepatocellular carcinoma) to confirm the MVI status of the resected tumor.

### The Volume of Interest Delineation

**Figure 1:**
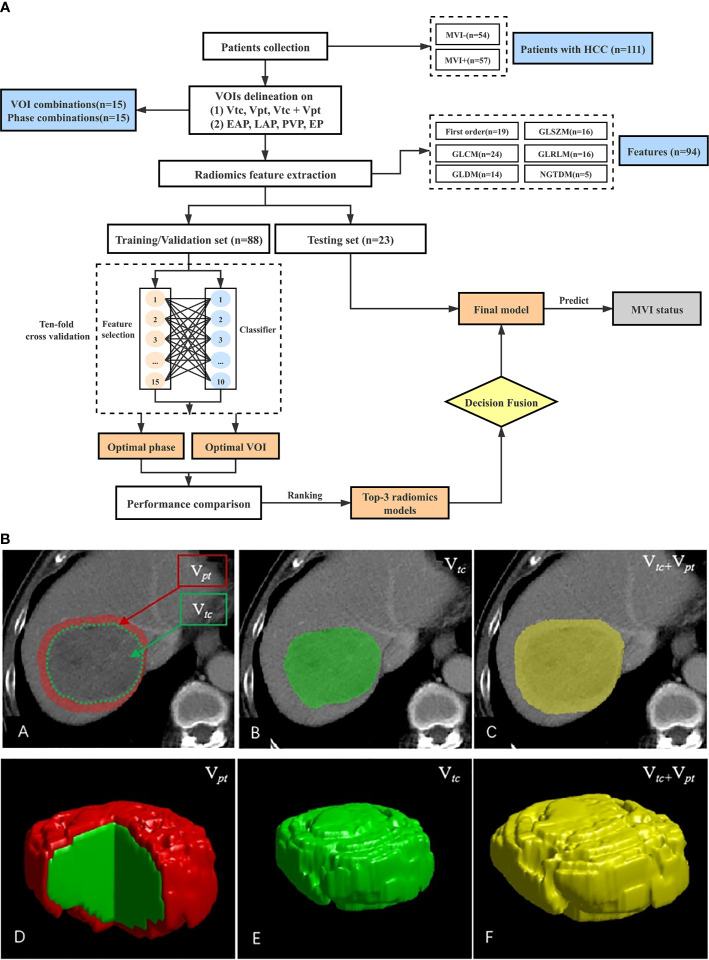
**(A)** Study workflow. **(B)** Lesion VOIs delineation illustrated in 2D (first row) and 3D (second row). The V*_tc_* (green) is the tumor core; the V*_pt_* (red) is the peritumor region at a given distance (2; 4; 6; 8; 10; 12; 14 mm) away from the tumor margin; the V*_tc_* + V*_pt_* (yellow) is the combination region of V*_tc_* and V*_pt_*. V*_tc_*, volume of tumor core; V*_pt_*, peripheral tumor regions; V*_tc_* + V*_pt_*, combination of V*_tc_* and V*_pt_*.

All images in each phase were stored in DICOM format and anonymized. Delineation of the target volume of interest (VOI) was performed by the ITK-SNAP software (http://www.itksnap.org) on the CT images slice-by-slice on phases 1- 4 (**Figure 1**). Visible tumor margins were first manually delineated to obtain the volume of tumor core (V*_tc_*). This procedure was conducted by two investigators (W.L Zhang and R.M Yang, with 4 and 15 years of experience in radiological diagnosis, respectively) who lacked prior knowledge of the patients’ MVI status. The conformity of delineated VOIs was measured by the Dice similarity coefficient. The two delineated VOIs with Dice index greater than 0.9 were averaged to yield the final VOI. Discrepancies on the lesion boundary (Dice < 0.9) were resolved by further discussions until mutual consensus were reached. The V*_tc_* was then extended to different distances (2; 4; 6; 8; 10; 12; 14mm) from the tumor margin, to obtain seven VOIs of the tumor periphery (V*_pt_*), which were automatically generated with a morphological dilation algorithm. This process was not entirely isotropic as the expansion would stop on encountering large vessels (vessel caliber ≥2mm), bile ducts, or liver margin. All the manual steps allowed slight adjustment to acquire tailored VOIs for each phase. The VOI delineation was performed on the largest lesion for patients with multiple lesions.

### Radiomics Feature Extraction and Analysis

Radiomic features were extracted from each VOI using an open-source python package Pyradiomics (https://pyradiomics.readthedocs.io/en/latest/index.html). There were 94 features in total extracted from the candidate features set including: 1) First order features (*n*=19); 2) gray level co-occurrence matrix (GLCM) features (*n*=24); 3) gray level size zone matrix (GLSZM) features (*n*=16); 4) gray level run length matrix (GLRLM) features (*n*=16); 5) neighboring gray-tone difference matrix (NGTDM) features (*n*=5); and 6) gray level dependence matrix (GLDM) features (*n*=14). Please refer to the Pyradiomics documentation (32–34) for their detailed definitions. Feature extractions were performed on each of the four phases, and the corresponding obtained features were combined and categorized into four groups:

1. *group 1*: features from each phase, termed as $F_{pha}^{1},\;F_{pha}^{2},\;F_{pha}^{3},\;and\;F_{pha}^{4}$ (*n*=94 features for each of the four types);
2. *group 2*: concatenated features of any two phases, $F_{pha}^{1;2},\;F_{pha}^{1;3},\;F_{pha}^{1;4},\;F_{pha}^{2;3},\;F_{pha}^{2;4},\;and\;F_{pha}^{3;4}$ (*n*=188 features for each of the six types);
3. *group 3*: concatenated features of any three phases, $F_{pha}^{1;2;3},\;F_{pha}^{1;2;4},\;F_{pha}^{1;3;4},\;and\;F_{pha}^{2;3;4}$ (*n*=282 features for each of the four types);
4. *group 4*: concatenated features of all four phases, $F_{pha}^{1;2;3;4}$ (*n*=376 features) (31).

This resulted in a total of 15 types (4 + 6 + 4 + 1) of different combinations of features, which then served as the input for a specific predictive model.

### Multi-Disciplinary Team-Like Prediction Modeling

The patient samples were divided chronologically into a training/validation set (*n* = 88) and an independent testing set (*n* = 23). In this research, a typical prediction model was developed on a feature selection strategy and a classifier, and was then cross-validated by a tenfold cross-validation (CV) using the training/validation set (90% training, 10% validation). In each step of the ten-fold CV, a specific feature selection method screened out an optimal subset of the features to train a particular classifier. Fifteen feature selection methods and ten classifiers were investigated and their possible combinations resulted in 150 different prediction models.

The 15 types of features were extracted from different combinations of VOIs (V*_tc_*, V*_pt_* or V*_tc_* +V*_pt_*, note here V*_pt_* is obtained with seven different tumor periphery distances), and then were fed into each of the above mentioned 150 models. We yield totally 33750 (15×150×(1 + 7+7)) models to be compared on the training/validation set. The predictive powers of all the evaluated models were quantified by the area under the receiver operating characteristic (ROC) curve (AUC), accuracy (ACC), sensitivity (SEN), and specificity (SPE).

The best phase and V*_pt_* for MVI status prediction were determined by comparing the AUC values. The top-3 ranked models were identified by ten-fold CV on the training/validation set from the best phase and V*_pt_*. These top-3 ranked models were then fused by two fusion methods, i.e., the plurality voting (PV) and the weighted fusion (WF), to generate the final model that was then verified on the independent testing set.

Each of the top-3 predictive models was regarded as a clinical specialist providing a prediction of MVI. The PV gives a consensus prediction based on the highest number of votes. PV counts the number of decisions for each class and assigns the sample *x_i_* to class *y_g_* which obtained the highest number of votes. All the classifiers have the same weight regardless of their respective abilities pertaining to effective classification. The final prediction result is calculated as $H(x)=arg\underset{j}{\max}\Sigma_{i}^{L}c_{i}^{j}(x_{i})$, where the $c_{i}^{j}(x)$ is the i-th classifier c_i_ output on the j-th class *y_g_* and L is the number of classifiers. Simply, the PV treats each vote with equal weight, to assume that each specialist has an equal contribution to the final prediction. While the WF assigns different weights to each classifier and integrates the classification results by a linear weighted summl: $H(x)=\Sigma_{i}^{L}w_{i}c_{i}(x)$, where *w_i_* is the assigned weight to classifier *c_i_*(*x*) satisfying *w_i_* > 0 and $\Sigma_{i}^{L}w_{i}c_{i}(x)$. In this paper, the *w_i_* is calculated based on the validation accuracy *acc_i_* of the i-th classifier computed during the training stage: $w_{i}=\frac{acc_{i}}{\Sigma_{k=1}^{L}\;acc_{k}}.$

The gain from model fusion was assessed by the metric: *Net Reclassification Improvement* (NRI), which is a quantitative measure for evaluating improvements in risk predictions from diagnostic tests and prediction models (**Supplementary Materials**) (35, 36).

### Statistical Analysis

All statistical analyses were conducted using the SPSS 25.0 software (IBM SPSS Corporation, USA) and python 3.6.2 (Python Software Foundation (USA, https://www.python.org/downloads/). Baseline patient characteristics were analyzed *via* univariate analysis. The categorical variables were presented as numbers and proportions and analyzed *via* the Chi-square test. Two-sided *p* values less than 0.05 were considered statistically significant. Comparisons between the 15 feature types were conducted using independent samples Kruskal-Wallis test with Bonferroni correction for adjusting for significant level in pairwise comparison.

## Results

### Demographics

The study cohort comprised of 57 MVI-positive and 54 MVI-negative patients who met the inclusion criteria. No statistically significant differences were seen in age, sex, presence of cirrhosis, hepatitis B and C virus infection, serum AFP, WBC, RBC, neutrophil count, Hb, ALB, PLT, PT, INR, AST, ALT, CB, TB, Scr, ALP, and Child-Pugh class in the training/validation and independent testing set (**Table 1**).

**Table 1:** Demographics and clinical characteristics.

Variable	Training/Validating set			Testing set		
	MVI−(*n* = 41)	MVI+(*n* = 47)	*p* values*	MVI−(*n* = 13)	MVI+(*n* = 10)	*p* values*
**Age**						
0, ≤50 years	8(19.5)	21(44.7)		3(23.1)	0(0)	
1, >50 years	33(80.5)	26(55.4)	0.012	10(76.9)	10(100)	0.103
**Sex**						
0, Female	5(12.2)	6(12.8)		1(7.7)	1(10)	
1, Male	36(87.8)	41(87.2)	0.936	12(92.3)	9(90)	0.846
**Hepatitis virus infection (HBV/HCV)**						
0, absent	7(17.1)	8(17.0)		4(30.8)	3(30)	
1, present	34(82.9)	39(83.0)	0.995	9(69.2)	7(70)	0.968
**Liver cirrhosis**						
0, absent	26(63.4)	22(46.8)		6(46.2)	3(30)	
1, present	15(36.6)	25(53.2)	0.119	7(53.8)	7(70)	0.722
**AFP**						
0, ≤20 µg/L (0–7.5)	17(41.5)	19(40.4)		3(23.1)	2(20)	
1, ≤400 µg/L (0–7.5)	11(26.8)	9(19.2)		4(30.8)	2(20)	
2, >400 µg/L (0–7.5)	13(31.7)	19(40.4)	0.597	6(46.1)	6(40)	0.785
**WBC**						
0, ≤10 × 10^9^/L	38(92.7)	42(89.4)		11(84.6)	9(90)	
1, >10 × 10^9^/L	3(7.3)	5(10.6)	0.719	2(15.4)	1(10)	0.704
**Neutrophil**						
0, ≤6.3 × 10/L	37(90.2)	40(85.1)		11(84.6)	9(90)	
1, >6.3 × 10^9^/L	4(9.8)	7(14.9)	0.467	2(15.4)	1(10)	0.704
**RBC**						
0, ≤3.8^a^/4^b^ × 10^9^/L	9(21.9)	5(10.6)		2(15.4)	4(40)	
1, >3.8^a^/4^b^ × 10^9^/L	32(78.1)	42(89.4)	0.148	11(84.6)	6(60)	0.393
**Hb**						
0, ≤128 g/L	13(31.7)	15(32.0)		3(23.1)	2(20)	
1, >128 g/L	28(68.3)	32(68.0)	0.983	10(76.9)	8(80)	1
**PLT**						
0, ≤100 × 10^9^/L	5(12.2)	4(8.5)		2(15.4)	1(10)	
1, >100 × 10^9^/L	36(87.8)	43(91.5)	0.728	11(84.6)	9(90)	1
**PT**						
0, ≤13 s	40(97.6)	45(95.7)		12(92.3)	8(80)	
1 >13 s	1(2.4)	2(4.3)	1	1(7.7)	2(20)	0.807
**INR**						
0, ≤1.0	35(85.4)	36(76.6)		11(84.6)	7(70)	
1, >1.0	6(14.6)	11(23.4)	0.299	2(15.4)	3(30)	0.739
**AST**						
0, ≤40 U/L	23(56.1)	23(48.9)		7(53.8)	5(50)	
1, >40 U/L	18(44.9)	24(51.1)	0.502	6(46.2)	5(50)	1
**ALT**						
0, ≤50 U/L	30(73.2)	37(78.7)		9(69.2)	7(70)	
1, >50 U/L	11(26.8)	10(21.3)	0.542	4(30.8)	3(30)	1
**DBIL**						
0, ≤6.8 µmol/L	29(70.7)	34(72.3)		9(69.2)	9(90)	
1, >6.8 µmol/L	12(29.3)	13(27.7)	0.867	4(30.8)	1(10)	0.492
**TBIL**						
0, ≤20 µmol/L	32(78.1)	31(66.0)		9(69.2)	6(60)	
1, >20 µmol/L	9(21.9)	16(34.0)	0.21	4(30.8)	4(40)	0.985
**ALP**						
0, ≤125^a^/135^b^ U/L	31(75.6)	33(70.2)		11(84.6)	9(90)	
1, >125^a^/135^b^ U/L	10(24.4)	14(29.8)	0.571	2(15.4)	1(10)	1
**Scr**						
0, ≤133 µmol/L	38(92.7)	45(95.7)		13(100)	10(100)	
1, >133 µmol/L	3(7.3)	2(4.3)	0.661	0(0)	0(0)	/
**ALB**						
0, ≤40 g/L	16(39.0)	17(36.2)		1(7.7)	3(30)	
1, >40 g/L	25(60.1)	30(63.8)	0.783	12(92.3)	7(70)	0.398
**Child–Pugh**						
0, A	36(87.8)	40(85.1)		12(92.3)	9(90)	
1, B/C	5(12.2)	7(14.9)	0.713	1(7.7)	1(10)	1

Unless indicated otherwise, data are numbers with percentages in the parentheses. HBV, hepatitis B virus; HCV, hepatitis C virus; AFP, serum alpha-fetoprotein; WBC, White blood cell; RBC, Red blood cell; Hb, Hemoglobin; PLT, platelet count; PT, prothrombin time; INR, international normalized ratio; AST, aspartate aminotransferase; ALT, alanine aminotransferase; DBIL, Direct bilirubin; TBIL, total bilirubin; ALP, Alkaline phosphatase; Scr, serum creatinine; ALB, serum albumin. *Chi-square test; ^a^female; ^b^male. A value of p ˂ 0·05 was considered statistically significant.

### Radiomics Analysis

#### Optimal Setting Determination

**Figure 2:**
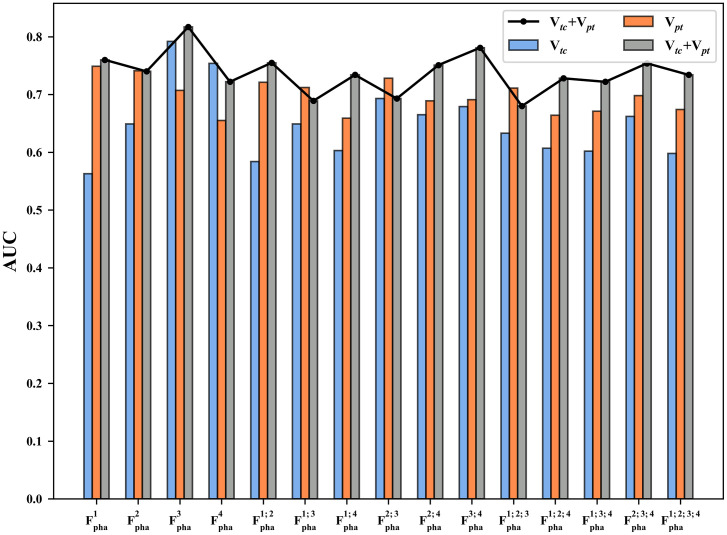
Prediction performance in terms of AUC on 15 phase combinations $\left(F_{pha}^{1},\;F_{pha}^{2},\ldots ,F_{pha}^{1;2;3;4}\right)$ and 3 VOIs combinations (V*_tc_*, V*_pt_* and V*_tc_* + V*_pt_*). V*_tc_*, volume of tumor core; V*_pt_*, peripheral tumor regions; V*_tc_* + V*_pt_*, combination of V*_tc_* and V*_pt_*; AUC, area under the ROC curve.

All the established predictive models were comprehensively compared to determine the optimal phases, tumor periphery, and VOIs combinations. **Figure 2** shows the prediction comparison results on 15 phase combinations $\left(F_{pha}^{1},\;F_{pha}^{2},\ldots ,F_{pha}^{1;2;3;4}\right)$ and three VOIs combinations (V*_tc_*, V*_pt_* and V*_tc_* +V*_pt_*). The AUCs were the mean values averaged for all the seven tumor peripheral distances and all the 150 classifiers. The best performance was seen in $F_{pha}^{3}$ (portal venous phase, PVP) and this conclusion was consistent for both V*_tc_* (AUC=0.78) and V*_tc_* +V*_pt_*(AUC=0.82). Models inclusive of the PVP phase had the second ($F_{pha}^{3;4}$ with V*_tc_*+ V*_pt_*, AUC = 0.78) and third-best ($F_{pha}^{2;3;4}$ with V*_tc_*+V*_pt_*, AUC=0.76) performances. In terms of VOIs combinations, the V*_pt_* generally achieved better predictive performance than the V*_tc_* in almost all phasic combinations (except for in $F_{pha}^{3}\;andF_{pha}^{4}$). The V*_tc_* +V*_pt_* had improved prediction as compared with V*_tc_* or V*_pt_* alone in most of the 15 phase. combinations (except in $F_{pha}^{2},\;F_{pha}^{4},\;F_{pha}^{1;3},\;F_{pha}^{2},\;F_{pha}^{2;3},\;F_{pha}^{1;2;3}$).

**Figure 3:**
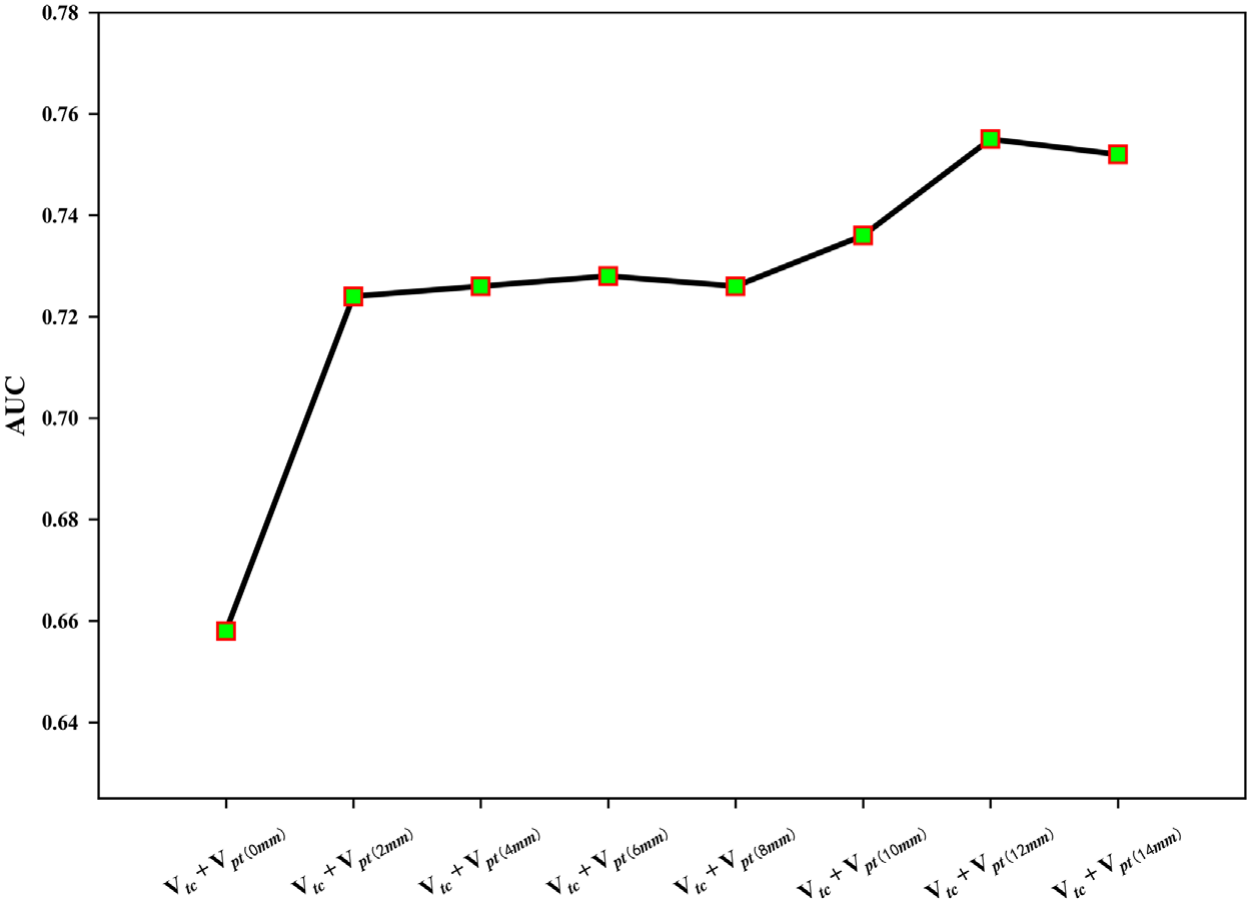
The prediction accuracy (AUC) trend with respect to increasing peripheral distances in V*_tc_*+ V*_pt_* (with $\;F_{pha}^{3}$).

Using the optimal phase $\left(F_{pha}^{3}\right)$ and VOIs combination (V*_tc_*+ V*_pt_*), the optimal tumor peripheral distance was determined by comparing the predictions on $F_{pha}^{3}$ and V*_tc_*+V*_pt_* with the aforementioned seven different peripheral distances. As shown in **Figure 3**, the predictive accuracy (in terms of the mean AUC averaged over all the 150 classifiers) increased gradually (maximal at 12mm) as larger tumor peripheral distance was involved.

### Microvascular Invasion Prediction Performance

The training/validation set was cross-validated with features from $F_{pha}^{3}$ and V*_tc_*+V*_pt_* (with 12mm tumor peripheral distance) on the 150 prediction models (classifiers + feature selection). The performances of all models were ranked, and the top-3 models were respectively combinations of “Random forest” & “t_score”; “Random forest” & “f_score” and “k-Nearest Neighbor” & “f_score”.

**Figure 4:**
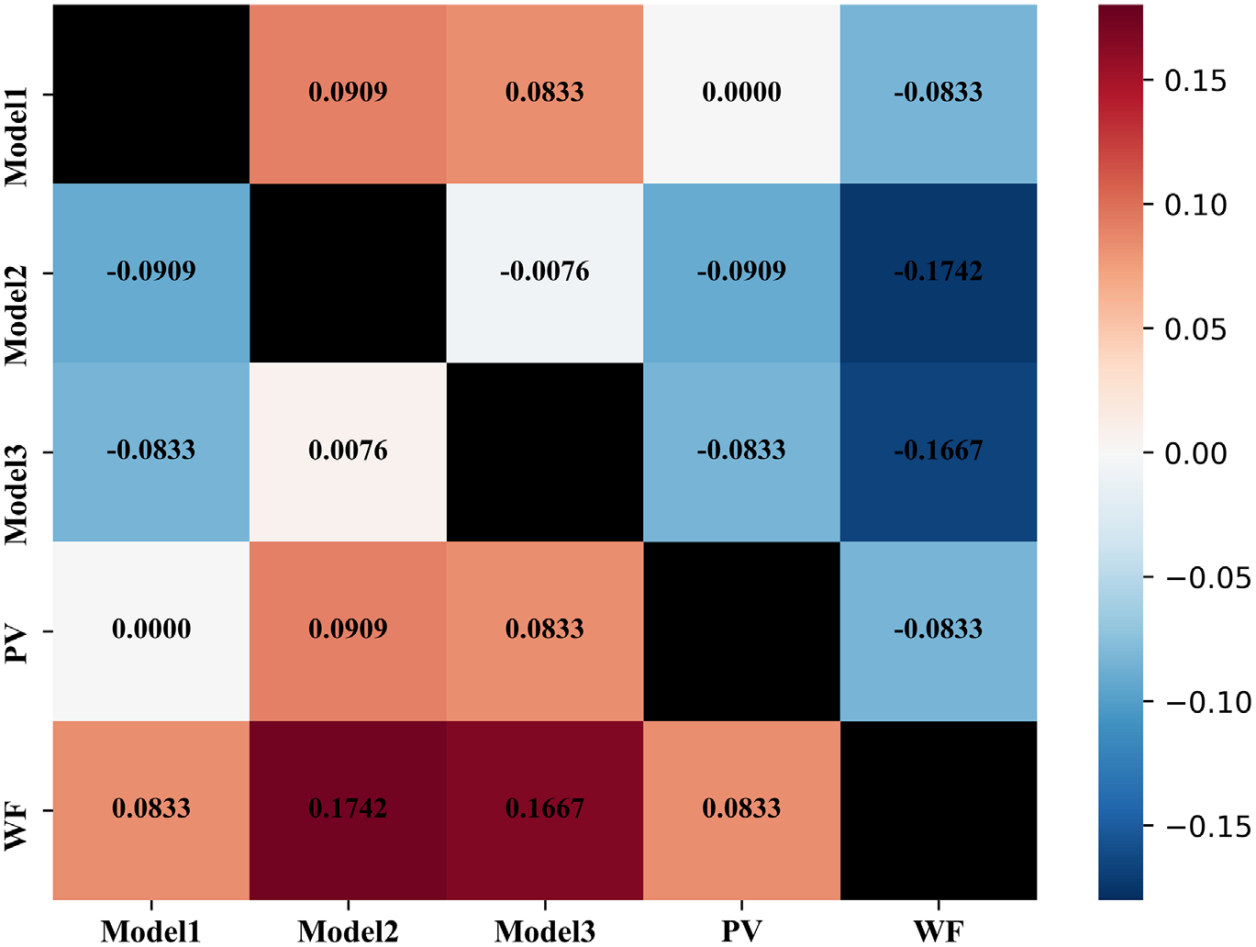
Quantitative comparisons of NRI between different models. Positive (or negative) NRI value indicates superiority (or inferiority). The NRI value in each cell represents the superiority (or inferiority) of a model in the *y*-axis to a model in the *x*-axis. NRI, Net Reclassification Improvement.

The predictive performances in terms of AUC of the top-3 models were 0.788, 0.776, and 0.775 on the training/validation set (with ten-fold CV), and 0.792, 0.78, and 0.803 respectively on the independent testing set (**Table 2**). Fusion of the top-3 models further improved the predictive accuracy to AUC = 0.795 with the PV method and AUC=0.811 with the WF method. Comparison between the top-3 models and the two fusion methods were quantified by the NRI metric, with a positive NRI value indicating superiority. It was observed that PV or WF fusion outperformed prediction using any of the top-3 models (lower-left corner in **Figure 4**).

**Table 2:** Predictive performances of the top-3 models and their fusion on the training/validation and independent testing sets.

Classifier + feature selection	Training/Validation set (*n* = 88)				Testing set (*n* = 23)			
	AUC	ACC	SEN	SPE	AUC	ACC	SEN	SPE
Randomforest + trace_ratio	0.788	0.704	0.70	0.67	0.792	0.739	**0.818**	0.667
Randomforest + f_score	0.776	0.684	0.65	0.65	0.78	0.70	0.727	0.667
k-Nearest Neighbor + f_score	0.775	0.70	0.784	0.61	0.803	0.70	0.818	0.583
Ensemble Methods	PV				0.795	0.739	**0.818**	0.667
	WF				**0.811**	**0.783**	**0.818**	**0.75**

The highest values are marked in bold.

### Top-Ranked Features

**Figure 5:**
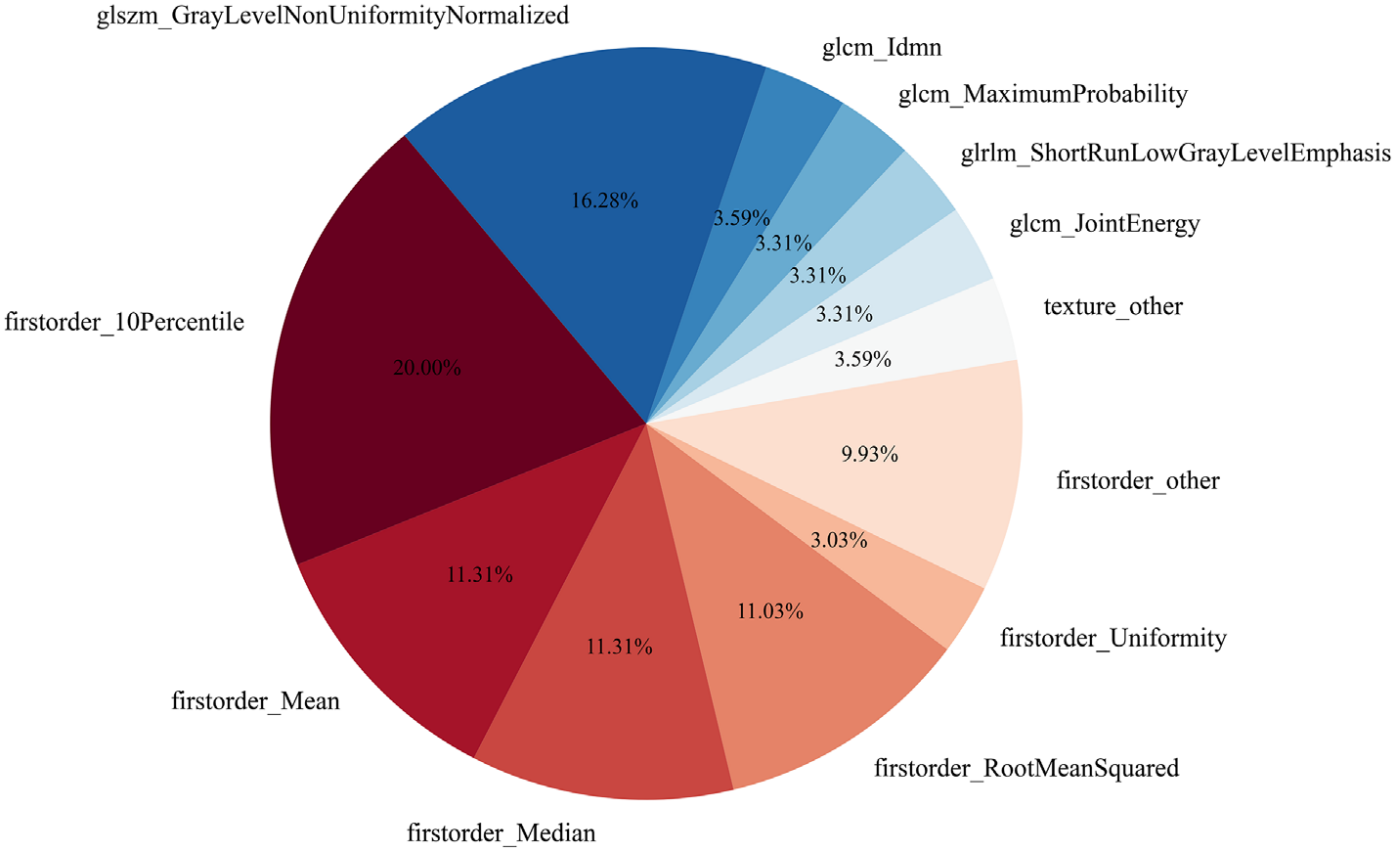
A pie chart showing the number of times (%) of the features in $\;F_{ph}^{3}$ being selected into the top 10 features in the 10-fold cross-validation of all predictive models with AUC > 0.7.

We also counted the number of times for each feature (with $F_{pha}^{3},$ V*_tc_*+V*_pt(12mm)_*) being selected as the top features in the 150 prediction models using ten-fold CV (**Figure 5**). The five most frequently selected features included: four first-order features (10^th^ percentile (20%), mean (11.31%), median (11.31%) and root mean square values (11.03%)) as well as one texture feature GLSZM-based Gray Level Non-uniformity Normalized (GLNN) (16.28%).

## Discussion

Image features extracted from the portal venous phase (PVP) with V*_tc_*+V*_pt_*_(12mm)_ were shown to be the most reliable features for MVI prediction in HCC. An AUC of 0.81 on the independent testing set was achieved by the WF fusion method by integrating the top-3 models ranked from the training/validation set. Also, one GLSZM-based texture feature GLNN and four first-order features were found to be most associated with MVI.

Previous studies using conventional CT/MR imaging reported the common manifestations indicative of positive MVI, as pseudo-capsule, unsmooth tumor margins, rim or peritumoral enhancement in the arterial phase, and peritumoral hypointensity in the hepatobiliary phase. Chou et al. reported that unsmooth tumor margin which has been confirmed to be correlated with pathologically extra nodules, multinodular fusion, or infiltrative margin, is indicative of positive MVI in HCC lesions. A correlation between focal extra nodules on CT images and MVI in the pathologic specimens was also found (20). Research work by Matsui et al. suggested that peritumoral regions had a concentration of tumor drainage vessels which presented as corona enhancement in CT hepatic arteriography (CTHA) and CT arterio-portography (CTAP) (37). Nishie et al. reported that MVI positive HCCs, especially those with lesion diameter < 3 cm, tended to have larger area of peritumoral enhancement (due to peritumoral hemodynamic change) than MVI negative HCCs (38). Although conventional radiological manifestations are known to provide hints regarding MVI status in HCC, inconsistent results have been reported in previous studies (perhaps owing to inter-observer variations) and thus standard diagnostic consensus has not been reached (16, 20).

Indeed, traditional radiological imaging analysis can be integrated with radiomics analysis for prediction modeling. However, radiographic diagnosis confirmed by naked-eye observation is usually limited by one’s visual perception, which is insensitive to subtle image differences. Furthermore, diagnostic performance of traditional radiological imaging analysis is closely related to the radiologist’s clinical experiences and can be easily biased by subjectivity. While radiomics provides an auxiliary alternative for radiologists to explore more hidden image patterns in characterizing diseases. Several studies have attempted to predict MVI status *via* radiomics analysis on DCE-CT or MRI (26–28, 30, 39, 40). For instance, Ma et al. extracted textural/non-textual features from the DCE-CT arterial phase (AP), PVP, and delay phase (DP) of the tumor core for MVI prediction. The PVP-based radiomics model was reported to achieve an AUC of 0.783 and 0.793 in the training and validation datasets, respectively (27). Similarly, Xu et al. compared radiomic features from the entire-volumetric interest (VOI_entire_), 50% of the entire tumor volume (VOI_50%_) and a 5mm annular region neighboring the tumor surface (VOI_penumbra_). It was shown that VOI_entire_ and VOI_penumbra_ (with AUC 0.841 and 0.829 in the training and validation sets respectively) outperformed VOI_50%_ in MVI prediction, which was consistent with this study (28). However, the combinational effect of VOI_entire_ +VOI_penumbra_ was not addressed. Nebbia et al. used multi-parametric MRI radiomics to predict MVI from the tumor core, at a fixed (10mm) peritumoral region, as well as the tumor core + peritumoral region (39). However, the fixed 10mm peritumoral margin was heuristically proposed which may omit MVI occurring in regions beyond this distance. The HCC pathological specimen collection standard may additionally support this as it includes liver tissue within a 2cm range from the tumor margin (6).

Previous investigations were conducted on a single (or two) DCE CT/MRI AP, PVP, or DP phase(s) and have not addressed the combination of phases (26–28). This study is the first attempt, to our knowledge, to provide a comprehensive analysis aimed at substantiating the predominant role of the PVP phase (27). More diagnostic attention should be emphasized on PVP. Also, EAP had limited contributions to MVI prediction. Exemption of an EAP scan is therefore recommended in routine enhanced CT to reduce the radiation exposure.

One GLSZM-based feature GLNN and four first-order features were shown to exhibit strong predictive capabilities of MVI. The GLNN measures the variability of gray-level intensity values in the image, with a lower value indicating greater similarity in intensity values (41). Our results implied that the MVI positive HCCs are associated with more heterogeneity within the V*_tc_*+V*_pt(12mm)_* than the MVI negative HCCs. This can be explained by the underlying HCC hemodynamic mechanism, that is, tumor cells may implant to the surrounding normal liver parenchyma from the tumor-draining vessels (37, 38). Furthermore, the MVI positive groups demonstrated higher 10^th^ percentile, mean, median and root mean square values. Perhaps, this may be attributed to the hyper-attenuation resulting from excretion of CT contrast agent *via* the tumoral drainage vessels to the surrounding normal liver parenchyma in PVP.

This study also employed a novel method of using classifier fusion for MVI prediction modeling, such as a process analogous to disease diagnosis by a MDT, produce more reliable and accurate predictions compared with a single classifier. This was based on two reasons: 1) First, there is large performance differences between different classifiers, which has been confirmed in our previous study (31). It is not practical to select a suitable classifier for a given task from a large pool of classifiers, since different classifiers are built on different mathematical grounds; 2) Second, fusion of classifiers has been proved to generate more stable and reproducible classification performance than an individual classifier, and is effective in improving classification/prediction accuracy in decision-making (32, 32, 42–44).

## Limitations

Our study has several limitations. First, this was a retrospective and single-center study with a relatively small sample size due to an inclusion requirement of the EAP phase. However, EAP is seldom performed in the local institutions. Future investigations should thus include more participants across different centers to confirm this study’s findings. Secondly, manual VOI delineation is time-consuming and has uncertainties on subsequent radiomics analysis and prediction modeling (45). Semi- or automatic segmentation methods are expected to generate more consistent and reproducible results.

## Conclusion

In conclusion, this study demonstrated the feasibility of noninvasive MVI prediction *via* CT radiomics analysis and a MDT-like fusion-based radiomics prediction modeling. Image features extracted from the portal venous phase on the tumor core and within 12 mm of the tumor peripheral region may be considered as potential quantitative imaging biomarkers.

## Data Availability Statement

The original contributions presented in the study are included in the article/**Supplementary Material**. Further inquiries can be directed to the corresponding authors.

## Ethics Statement

Written informed consent was obtained from the individual(s) for the publication of any potentially identifiable images or data included in this article

## Author Contributions

WZ, RY, and FL conducted the literature search. WZ, RY, XW, XZ, and XJ designed the study. WZ, GL, WD, and HW collected the data. WZ, RY, XZ, FL, SL, and AC analyzed the data. All authors verified the data. WZ, RY, FL, SL, XZ, and XJ edited the manuscript. RY, XW, XZ, and XJ reviewed the manuscript. All authors contributed to the article and approved the submitted version.

## Funding

This study received funding from the National Natural Science Foundation of China (81971574, 81874216, and 81571665), the National Key Research and Development Program of China (2017YFC0112900), the Natural Science Foundation of Guangdong Province, China (2018A030313282), the Guangzhou Science and Technology Project, China (202002030268 and 201904010422), and the Medical Science and Technology Research Project of Guangdong Province (A2019465).

## Conflict of Interest

The authors declare that the research was conducted in the absence of any commercial or financial relationships that could be construed as a potential conflict of interest.
