# Supplementary material for: Prediction of Microvascular Invasion in Hepatocellular Carcinoma With a Multi-Disciplinary Team-Like Radiomics Fusion Model on Dynamic Contrast-Enhanced Computed Tomography
Source: Front Oncol. 2021 Mar 16;11:660629. doi: 10.3389/fonc.2021.660629 (PMC8008108; doi:10.3389/fonc.2021.660629)
Supplement: Supplementary file 1 [file DataSheet_1.doc]

**Supplementary materials:**

***DCE CT imaging parameters***

Preoperative DCE-CT images were obtained on multiple scanners: a 320-slice spiral CT scanner (Siemens Medical Solutions), Philips Brilliance 64, and Toshiba Aquilion One 16. The scanners had the following parameters: tube voltage120 kVp, 200 mAs, and a reconstruction slice thickness of 5 mm. After collection of unenhanced images, all patients were injected with intravenous nonionic iodinated contrast agent (iodipamide, 370 mg I/mL, Bracco) via the antecubital vein by mechanical power injectors based on their weight (2.0 mL/kg body weight, with a maximal dose of 180ml), followed by a 20-mL saline flush. Finally, four-phase CT scans images were obtained: phase 1, early arterial phase (EAP, 18-25 s); phase 2, late arterial phase (LAP, 35-40 s); phase 3, portal venous phase (PVP,50-60 s), and phase 4, equilibrium phase (EP,120-250 s).

All the image data was standardized to have unanimous pixel spacing before feature extraction and subsequent model training.

***Net Reclassification Improvement***

The Net Reclassification Improvement (NRI) is used to quantify the differences between two models in the correct classification of the number of research objects, as well as improvement of one model over the other. The NRI is calculated as:

*NRI= [(*
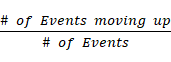
*) - (*
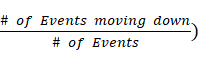
*] +[ (*
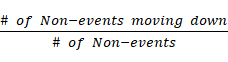
*) - (*
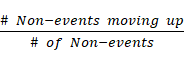
*)]*

#: the number of cases.

Events: cases in model 1.

Non - events: cases in model 2.

Moving up: cases correctly classified.

Moving down: misclassified cases.

NRI > 0, is an improvement, indicating that the predictive ability of model 1 was improved compared with model 2. NRI < 0, was negative, indicating that the predictive ability of model 1 was worse than model 2.

**Table:**

**Table S1. Feature selection methods and classifiers**

| **Feature selction methods**  (n=15) | DISR, JMI, fisher_score, lap_score, reliefF, SPEC, trace_ratio, ls_l21, MCFS, NDFS, RFS, CFS, f_score, gini_index, t_score |
| --- | --- |
| **Classifiers**  (n=10) | LogisticRegression(LR), K-nearest neighbors(KNN), Support vector machine(SVM), DecisionTree(DT), Naïve Bayes, RandomForest(RF), ExtraTrees(ET), Bagging, AdaBoosting, Gradient Boosting Decision Tree (GBDT) |
